# Cancer treatment-induced thrombocytopenia: diagnosis, mechanisms and management

**DOI:** 10.3389/fimmu.2025.1634688

**Published:** 2025-09-23

**Authors:** Xutong Zhao, Xiu Shan, Shaofeng Sui, Qinghao Song, Miao Cheng, Yi Zhao

**Affiliations:** First Affiliated Hospital, Dalian Medical University, Dalian, China

**Keywords:** CTIT, chemotherapy-induced thrombocytopenia (CIT), immune checkpoint inhibitors-induced thrombocytopenia (ICIIT), TPO-RAs, traditional Chinese medicine (TCM)

## Abstract

Cancer treatment-induced thrombocytopenia (CTIT) is a common adverse effect in malignant tumor patients, significantly increasing the risk of bleeding and negatively impacting treatment efficacy and quality of life. Current treatment options for CTIT primarily include platelet transfusion, recombinant human interleukin-11 (rhIL-11), recombinant human thrombopoietin (rhTPO) and thrombopoietin receptor agonists (TPO-RAs). However, these methods have their limitations; for instance, platelet transfusions may cause adverse reactions, and the efficacy and safety of rhTPO and TPO-RAs remain controversial. This review aims to summarize the current treatment landscape for CTIT and explore new therapeutic advancement, including the potential role of traditional Chinese medicine, in order to provide more effective treatment strategies for clinical practice.

## 1 Introduction

CTIT is defined as a reduction in platelet count that occurs as a consequence of anti-tumor therapies, significantly increasing the risk of bleeding and negatively impacting treatment efficacy and quality of life. The definition of CTIT refers to platelets in peripheral blood (PLT) counts below 100×10^9^/L caused by antitumor drug therapy and includes CIT,ICIIT and targeted therapy-induced thrombocytopenia (TTIT). Research has shown that CTIT can affect up to 21.8% of patients undergoing antitumor therapy, particularly in hematological malignancies and solid tumors treated with myelosuppressive drugs (1). This condition is clinically significant as it can lead to a range of complications, including increased risk of bleeding, delays in treatment, and a potential decrease in overall treatment efficacy. The incidence of CTIT varies depending on the type of cancer, the specific treatment regimen employed, and individual patient factors (2). Understanding CTIT is crucial for oncologists and healthcare providers, as its management can profoundly impact patient outcomes and quality of life.

The mechanisms behind CTIT are multifaceted and often involve direct bone marrow suppression due to cytotoxic agents, immune-mediated mechanisms, or the effects of the tumor microenvironment (3).Certain cancers, such as hematological malignancies, are more inclined to causing CTIT because of their inherent effects on blood cell production. Additionally, certain chemotherapy regimens used can exacerbate the situation of thrombocytopenia. For instance, regimens that include agents known for their myelosuppressive properties are more likely to result in significant drops in platelet count (4). In the context of cancer treatment, comprehensive summary of clinical prevalent therapeutic approaches to mitigate this adverse effect is crucial for enhancing our understanding and management of this unfavorable condition.

As such, the relationship between tumor type, treatment strategy, and the occurrence of CTIT is a critical area of research that seeks to optimize treatment protocols and minimize adverse effects. The impact of CTIT on patient care is significant, affecting not only the current treatment plan but also increase the need for frequent monitoring (5). In severe cases, patients may require platelet transfusions, which can complicate treatment regimens and increase healthcare costs (6). Therefore, addressing CTIT is essential not only for improving treatment efficacy but also for enhancing the overall well-being of patients undergoing anti-cancer therapy. This review aims to explore the mechanisms, implications, and management strategies associated with CTIT, providing a comprehensive overview for clinicians and researchers.

## 2 Diagnosis, clinical manifestations and epidemiology of CTIT

### 2.1 Laboratory tests and diagnostic criteria for CTIT

The clinical diagnosis for CTIT is indispensable to include a definite history of using a certain anti-tumor drug that may cause thrombocytopenia and the symptoms of thrombocytopenia gradually decrease or disappear after stopping the drug. Besides, other causes of thrombocytopenia have been excluded, such as aplastic anemia, acute leukemia, radiation sickness, immune thrombocytopenic purpura, bone marrow invasion by tumors, and hyperactive spleen function (7).The diagnosis of thrombocytopenia is primarily established through laboratory testing, which includes a complete blood count (CBC) that reveals the platelet count. A platelet count below 150,000 platelets per microliter is generally considered thrombocytopenia. Non-chemotherapy-related thrombocytopenia is generally diagnosed when the platelet count falls below 100 × 10^9^/L, which differs from the criteria for grade 1 CTIT. The grading criteria for CTIT is shown in **Table 1**. Further laboratory investigations may include peripheral blood smears to assess platelet morphology and the presence of any atypical cells, as well as specific tests to determine the underlying cause, such as bone marrow biopsy, immunological assays for autoimmune conditions, and viral serologic tests for infections like HIV or hepatitis (8, 9). Diagnostic criteria vary depending on the suspected etiology; for example, in ITP, the diagnosis may be supported by the exclusion of other causes of thrombocytopenia and the presence of specific clinical signs (10). Overall, a comprehensive approach combining clinical evaluation and targeted laboratory tests is essential for accurate diagnosis and management.

**Table 1:** CTIT grading criteria.

Grade	Platelet count (×10^9^/L)
Grade 1	<Lower Limit of Normal (LLN)* to 75
Grade 2	< 75 to 50
Grade 3	< 50 to 25
Grade 4	< 25
Grade 5	Death related to thrombocytopenia

*The Lower Limit of Normal (LLN) = 100×10^9^/L.

Early-detection biomarkers for CTIT can be grouped into four categories and is shown in **Table 2**: peripheral complete blood count–derived parameters, bone marrow–megakaryocyte kinetic indices, soluble protein/nucleic acid biomarkers, and imaging/functional indicators (11, 12). All of these can provide an early warning before the absolute platelet count falls below the critical threshold.

**Table 2:** Early-detection biomarkers for CTIT.

Biomarkers	Description
Peripheral complete blood count–derived parameters (already routinely available)	
Immature platelet fraction (IPF)	Reflect compensatory megakaryocyte activity in bone marrow; an IPF > 6.3–11.7% predicts a higher likelihood of subsequent grade ≥ 3 CIT.
Absolute immature platelet count (AIPC = IPF % × PLT)	A sustained decline in AIPC precedes the drop in platelet count by 3–5 days, serving as a sensitive indicator of impaired “production–consumption” balance.
Early platelet decline slope (ΔPLT-48 h)	A≥30% fall in platelets within 48 h after chemotherapy carries a > 80% positive predictive value for persistent CIT.
Bone marrow–megakaryocyte kinetic indices (require marrow aspiration or imaging)	
Megakaryocyte ploidy histogram	Flow-cytometric or imaging analysis showing < 20% 8N/16N polyploid megakaryocytes indicates early differentiation arrest.
Megakaryocyte Maturation Index (Mk-MI)	A decreased proportion of CD41^+^CD42b^+^ high-ploidy cells predicts CIT one cycle in advance.
Soluble protein and nucleic acid biomarkers (research-to-translation phase)	
Serum TPO level	A TPO level > 500 pg/mL on days 3–5 after chemotherapy shows a dose-dependent association with subsequent grade ≥ 3 CIT.
Myelosuppression-related microRNAs	Up-regulated miR-150-5p and miR-155-5p in platelet microparticles (PMPs) precede the platelet drop by 5–7 days.
Circulating megakaryocyte-derived extracellular vesicles (Mk-EVs)	Reduced Mk-EVs/CD61^+^ counts mirror increased megakaryocyte apoptosis, with 90% sensitivity and 85% specificity.
Imaging/functional indicators	
Dynamic contrast-enhanced CT (DCE-CT) bone-marrow perfusion parameters	A > 20% decrease in pelvic bone-marrow blood flow (BF) is significantly associated with platelet counts < 75 × 10^9^/L within the next 2 weeks.
Spleen length & Overall Myeloablative Injury Score(OMIS) score	Baseline spleen length > 100 mm or a high OMIS score increases the risk of CTIT by 2.3-fold.

### 2.2 The epidemiology of CIT

CIT’s prevalence varies significantly depending on the types of cancer and the specific chemotherapy regimens employed. CIT was most commonly observed in non-small cell lung cancer (NSCLC) (25%), ovarian cancer (24%), and colorectal cancer (18%) (2). Studies have shown that CIT can affect up to 50% of patients undergoing certain chemotherapy protocols, particularly in hematological malignancies and solid tumors treated with myelosuppressive drugs (13). A clinical study in the United States enrolled adult patients with solid tumors and hematological malignancies receiving chemotherapy to investigate the risk of thrombocytopenia in chemotherapy regimens. The overall incidence of CIT was 13%, grade 3 thrombocytopenia (PLT: 25-50×10^9^/L) and grade 4 thrombocytopenia (PLT: < 25 × 10^9^/L) was 4% and 2%,respectively. The incidence of thrombocytopenia varies by tumor type and chemotherapy regimen, with grade 3 thrombocytopenia occurring in approximately 4% of patients with solid tumors and 16% of patients with hematologic malignancies (14). Thrombocytopenia prevention can ensure that chemotherapy goes smoothly as planned and is beneficial to the long-term survival of patients. In a systematic review and meta-analysis which included 125 clinical trials and 20,128 patients, the incidence of ICIIT was 1.16%,82 patients occurred ICIIT-related deaths (15). The incidence of thrombocytopenia caused by chemotherapy drugs, targeted therapy drugs and immune checkpoint inhibitors (ICIs) is listed in **Table 3**.

**Table 3:** Incidence of common anti-tumor drugs related thrombocytopenia.

Drug classification	Regimen drug name	Tumor	Incidence (%)	Grade 3/4 (%)
Chemotherapy drugs	Gemcitabine (16, 17)	pancreatic cancer	50.4	3.6-4.5
	Gemcitabine+oxaliplatin (18)	pancreatic cancer		11
	Gemcitabine+ cisplatin/carboplatin (19)	non-small-cell lung cancer(NSCLC)		35.1
	Capecitabine (20)	gastric cancer	27.3	2.3
	Carboplatin (21)	advanced solid tumors		23
	Cisplatin (22)	carcinomas of unknown primary		4
	Pemetrexed+carboplatin (23)	NSCLC		24
	Etoposide+cisplatin (24)	small-cell lung cancer(SCLC)		16
	Irinotecan (25, 26)	colorectal cancer		1.1-3.9
Targeted therapy drugs	Dasatinib (27)	myeloid leukemia		23-34.9
	Imatinib (28)	gastrointestinal stromal tumors		1.3
	Fruquintinib (29)	colorectal Cancer	20.9	4
ICIs	Sintilimab+ chemotherapy (30)	gastric cancer	66.2	24.7
	Camrelizumab (31)	Hodgkin Lymphoma	13.3	1.3
	Toripalimab (32)	melanoma	7.8	1.6
	Tislelizumab	Hodgkin lymphoma	11.4	1.4

### 2.3 Clinical symptoms of thrombocytopenia

Thrombocytopenia present with a variety of clinical symptoms that are often indicative of its severity and underlying causes, not only characterized by reduced platelet count. Patients may experience easy bruising, prolonged bleeding from cuts and spontaneous bleeding, such as petechiae or purpura on the skin. In more severe cases, patients might present with significant hemorrhage, including gastrointestinal bleeding or intracranial hemorrhage, which can be life-threatening. The clinical manifestations can vary depending on the etiology of the thrombocytopenia. For instance, in cases of immune thrombocytopenic purpura (ITP), patients may exhibit more pronounced skin manifestations, while those with thrombocytopenia due to bone marrow suppression may show signs of anemia or leukopenia as well (4, 33). Additionally, patients with underlying conditions such as liver disease may present with coagulopathy, further complicating the clinical characterization. In addition to causing bleeding in skins and internal organs, CIT may also lead to a delay in subsequent chemotherapy cycles, dose reduction, treatment interruption, and a decrease in relative dose intensity (delivered dose intensity/standard dose intensity), thereby affecting therapeutic efficacy (34). Understanding these manifestations is crucial for timely diagnosis and management, as they can significantly affect patient outcomes. The median time to the first drop in platelet count is 1–2 weeks after chemotherapy; with certain agents, CIT may appear beyond 2 weeks (14). ICIIT typically occurs within 12 weeks after drug initiation, with a median onset of approximately 41 days (35, 36); however, it can arise at any time, even after treatment has been discontinued.

### 2.4 Influencing factors of CTIT

CTIT is characterized by decreased platelet count due to the cytotoxic effects of anti-tumor drugs, understanding the multifactorial causes of CTIT is essential for effective management and prevention strategies. The risk factors for CTIT are listed in **Table 4** and can be grouped into four dimensions—patient baseline, tumor, treatment, and comorbidities. All key points are derived from high-quality cohort studies published in 2024 and the most recent expert consensus (37).

**Table 4:** Risk factors of CTIT.

Category	Risk factors
Patient baseline	Advanced age (≥ 65 years)
	Poor performance status (ECOG ≥ 2)
	High Charlson Comorbidity Index (CCI ≥ 2)
	Baseline hepatic or renal dysfunction
	Low platelet count before anticancer therapy
	Prior history of myelosuppression
Tumor-related	Solid tumors (bone, gastricintestinal, respiratory, urinary, biliary, ovarian, etc.) confer higher risk than breast cancer
	Locally advanced or metastatic stage
	Bone-marrow infiltration or metastasis
Comorbidities	Chronic liver disease (cirrhosis) or autoimmune disorders
	Hypersplenism
	Disseminated intravascular coagulation (DIC)
	Sepsis/severe infection
	Nutritional deficiency: vitamin B12 or folate
	Baseline thrombotic risk requiring antiplatelet therapy
Treatment-related	
Chemotherapy regimens	Gemcitabine, platinum agents (cisplatin, carboplatin, oxaliplatin), anthracyclines, taxanes, fluoropyrimidines/capecitabine, pemetrexed, alkylating agents, topotecan, etc.
	High dose density, large cumulative dose, combination regimens
Radiotherapy	Irradiation fields encompassing pelvis, long bones, or total-body irradiation (TBI)
	Concurrent chemoradiation further increases risk
Targeted/immunotherapy	Tyrosine kinase inhibitors(TKIs) (e.g., sorafenib, sunitinib), PARP inhibitors, PD-1/PD-L1 inhibitors

A multivariable model regression analyses exhibited three predictive variables for CIT which includes high ferritin, estimated glomerular filtration rate(eGFR)<60ml/min/1.73m^2^ and body mass index (BMI)<23kg/m^2^,and the model owned good predictive accuracy (38). Among a comprehensive analysis of various clinical and laboratory indicators, tumor site, treatment line, AST levels, oxaliplatin, and capecitabine were identified as important factors that influenced the occurrence of CIT (39). In a retrospective study, serum potassium ion, serum lactate dehydrogenase, platelet count, red blood cell count, and estimated glomerular filtration rate were predictors of serious CIT (40). One study also reported that chemotherapy, bone or brain metastasis were risk factors for thrombocytopenia during radiotherapy (41). Renal function is an important factor affecting chemotherapy-related thrombocytopenia (38), and patients with inadequate renal function have a greater degree of thrombocytopenia and poorer tumor treatment outcomes. The reason is that improved renal function leads to enhanced elimination of chemotherapy drugs, thereby reducing the adverse effects on megakaryocytes.

## 3 The mechanisms of anti-tumor drugs induced thrombocytopenia

### 3.1 CIT

The occurrence of CIT is multifactorial, involving various mechanisms that disrupt normal hematopoiesis and platelet production (7). Chemotherapy drugs are known to not only target cancer cells but also exert a significant inhibitory effect on bone marrow hematopoiesis (42). The cytotoxic effects of chemotherapeutic agents can lead to reduction in the number of megakaryocytes, the precursor cells that give rise to platelets. Additionally, the bone marrow microenvironment may be altered by chemotherapy, leading to further impairment of hematopoietic function and exacerbating thrombocytopenia (43). Chemotherapy drugs can cause drug-induced immune thrombocytopenia, platelet antibodies can be detected in the blood of patients, resulting in increased destruction of platelets. Previous studies have reported that repeated use of oxaliplatin can induce and sustain immune responses, leading to immune thrombocytopenia (44–47). Oxaliplatin can cause injury to the hepatic sinusoidal endothelial cells, disrupting the sinusoidal barrier and subsequently leading to collagen deposition and veno-occlusive fibrosis. This damage increases intrahepatic vascular resistance and causes portal hypertension. Thrombocytopenia in this context is moderate but prolonged, typically appearing after about 18 weeks of treatment (46, 48). Due to splenomegaly, there is an increase in the sequestration and destruction of platelets within the spleen (49).

Thrombocytopenia was observed in 7%–25% of patients treated with a carboplatin-based regimens, which occurred more frequently than those receiving cisplatin-based regimens (50). When gemcitabine is used as a single-agent chemotherapy, the incidence of thrombocytopenia is approximately 3.4% to 12%. This incidence is significantly higher when used in combination chemotherapy regimens(such as gemcitabine/carboplatin) (51–54). Thrombotic thrombocytopenic purpura (TTP) is a rare but severe complication in the treatment of gemcitabine characterized by hemolysis, thrombocytopenia, renal insufficiency and neurological symptoms with an incidence of 0.015% to 1.4% (55). Anthracycline drugs exert their antitumor effects by intercalating into the DNA double helix, thereby inhibiting the synthesis of DNA and RNA. However, this mechanism also exerts toxicity on normal hematopoietic cells, particularly on megakaryocytes in the bone marrow, leading to a reduction in platelet production (56). During the metabolic process in the body, anthracycline drugs generate free radicals, which lead to oxidative stress and subsequently damage hematopoietic cells in the bone marrow and platelets (57). The incidence of grade 3 thrombocytopenia during bortezomib treatment for relapsed, refractory myeloma is relatively high, which is approximately 14.5% (58).

### 3.2 ICIIT

ICIs have become a breakthrough in cancer treatment. For patients with NSCLC, ICIs are generally safer and better tolerated compared to cytotoxic drugs. However, their immune-related adverse events (irAEs) are increasingly receiving concern in clinical practice, especially the hematological irAEs (haem-irAEs), which are infrequent but can be life-threatening (59). The incidence rate of immune-related thrombocytopenia (irTCP) is 1.16%, with 82 cases of death related to ICIIT (0.45%) (15, 60). When used alone, the incidence of thrombocytopenia with PD-1/PD-L1 inhibitors is only 0.2%, but when combined with other treatments, the incidence can significantly increase to 6% to 6.8% (61–63). Consequently, ITP has emerged as the most prevalent haem-irAE in immunotherapy. Intravenous immunoglobulin (IVIG) is a common method for treating primary immune thrombocytopenia (ITP). In the treatment of ITP, IVIG can temporarily block the destructive effects of antibodies on platelets, thereby increasing the number of platelets. The interval between treatments depends on the platelet count and the condition of the disease, usually once a week (63). In addition, IVIG is also used in other irTCP conditions, such as ICIIT, and can rapidly increase platelet count to a safe level, especially in emergency situations (64).

A retrospective study launched by Ohio State University Comprehensive Cancer Center reviewed patients treated with ICIs between January 2011 and June 2017. The results indicated that out of 1038 patients treated with ICIs, 89 (8.6%) developed grade 3 or 4 thrombocytopenia, and 18 (20%) were attributed to ICIs after manual review and exclusion of other causes (accounting for 1.73% of the entire cohort). Thrombocytopenia and leucopaenia occurred earlier than the other hem-irAEs among ICI-treated cases such as autoimmune hemolytic anemia, with a median time of 40 days, and the onset of haem-irAEs was earlier in patients treated with combination therapy than monotherapy (65). The incidence of irTCP seemed to be lowest among patients treated with PD-1/PD-L1 monotherapy (p = 0.059). Patients who developed grade 3 or higher irTCP had poorer overall survival (66). In previous case reports related to ICIIT, corticosteroids, immunoglobulins, and TPO-RAs were administered, with 5 cases showing improvement and 3 cases resulting in death (67). A retrospective study of 2,360 melanoma patients treated with ICIs showed that less than 1% of patients experienced thrombocytopenia, with the majority of these patients’ platelet count returning to normal on their own (68).

The number of reported ITP cases is the most common in nivolumab and pembrolizumab in a systematic review study, there were 125 cases of ITP reported with pembrolizumab and 160 cases reported with nivolumab,338 cases totaled for all drugs. Higher incidence of ITP cases associated with PD-1 inhibitors nivolumab and pembrolizumab in comparison with oxaliplatin was noticed, but similar reporting with the PD-L1 inhibitors atezolizumab, avelumab,and durvalumab. In the literature review, melanoma (35%) and non-small-cell lung cancer (39%) were identified as the predominant types of cancer reported ICIIT in the case studies (69).

**Figure 1:**
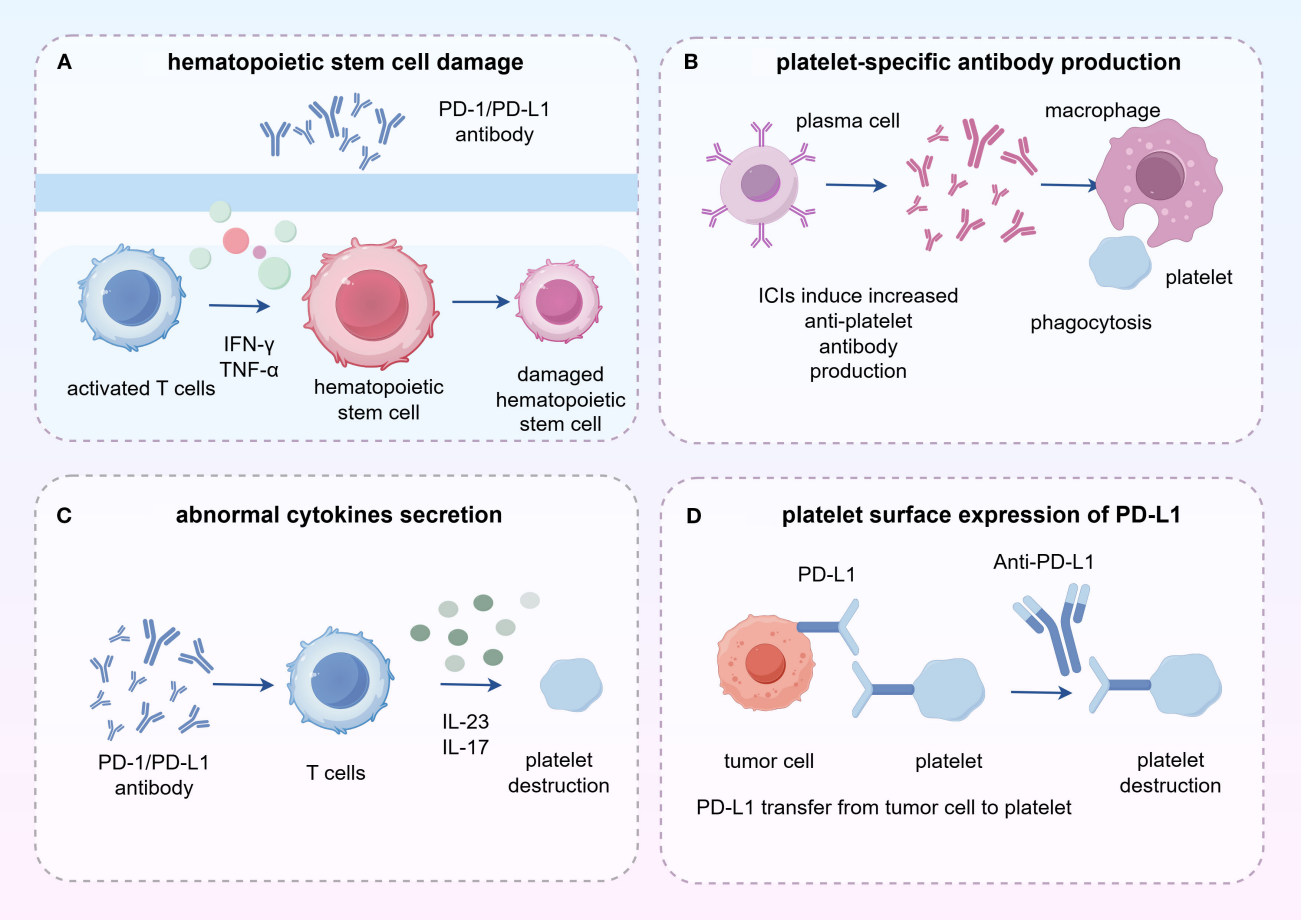
The mechanism of thrombocytopenia induced by ICIs.

Some mechanisms at the cellular level have been proposed in irTCP caused by anti-tumor immune therapy (65, 70) and is shown in **Figure 1**. ICIs such as PD-1 and PD-L1 inhibitors can cause immune system activation and exhausted CD4^+^ helper T cells and CD8^+^ cytotoxic T cells are reactivated (36). These activated T cells can secrete cytokines (such as IFN-γ, TNF-α, etc.) that lead to changes in the bone marrow microenvironment, thereby damaging hematopoietic stem cells and megakaryocytes (71). Cytokine storm induced by immunotherapy is a systemic inflammatory response that can be triggered by various causes, including infection, autoimmune diseases and adverse reaction to cancer immunotherapy (72).In the process of immunotherapy, cytokine release syndrome (CRS) is especially conscious of when using ICIs and immune cell therapies and relevant to adverse reaction to cancer immunotherapy (73). Cytokines manifest over-activating of the immune system which increases the incidence of irAEs after initiating ICI and serves as prediction of platelet count decrease (74). ICIs may induce or increase the production of specific anti-platelet antibodies, which can bind to platelets, marking them for phagocytosis and destruction by macrophages (75, 76). When patients have platelet antibodies in their bodies, the transfused platelets may be rapidly destroyed, leading to ineffective transfusion. The detection of platelet glycoprotein-specific auto-antibodies is a test with high specificity for antibody-mediated immune thrombocytopenia, which can differentiate between immune and non-immune thrombocytopenia.

Moreover, activated T cells secrete a large number of serum cytokines, such as IL-23 and IL-17,which can lead to reduced platelet production and increased platelet destruction. In immune thrombocytopenia (ITP), the levels of IL-23 and IL-17 are elevated (77). IL-17 plays a particularly important role in the production of inflammatory cytokines and the infiltration of white blood cells and can promote inflammatory responses of the immune system (78). IL-17 can inhibit the differentiation and maturation of megakaryocytes mediated by TPO, and affect platelet production and function, thereby further exacerbating the pathological process of ITP (79, 80). IL-23 can lead to reduced platelet production in ITP patients through the differentiation and proliferation of Th17 cells (81). Conversely, interleukin-6 (IL-6) is a multifunctional cytokine that plays an important role in immune responses, inflammatory reactions, hematopoiesis, and metabolic regulation (82). IL-6 can act synergistically with other cytokines to promote the growth of early bone marrow stem cells and enhance the differentiation of blood cells, including the production of platelets. IL-6 is a strong inducer of acute-phase proteins, which can induce hepatocytes to synthesize acute-phase proteins at the transcriptional level, including thrombopoietin (TPO), thereby indirectly affecting platelet production (83). The signaling pathways activated by IL-6 can affect the activation state and function of platelets, including platelet aggregation and adhesion.

Platelets expressing PD-L1 on their surface are direct target cells for ICIs. A study revealed that PD-L1 was transferred to platelets from tumor cells after co-incubated with various NSCLC cell lines, platelet PD-L1 (pPD-L1) was correlated with tumor stage, metastasis and overall survival (84). Platelets with high PD-L1 stimulated by atezolizumab mark improved immune surveillance and prevent T cell-mediated immune suppression (85).

### 3.3 TTIT

Targeted therapy has emerged as a revolutionary approach in oncology, focusing on specific molecular targets associated with cancer. Unlike traditional chemotherapy, which indiscriminately affects rapidly dividing cells, targeted therapies aim to interfere with particular pathways critical for cancer cell survival and proliferation. Targeted therapies are designed to attack specific cancer cells, can inadvertently affect normal hematopoiesis, leading to adverse effects such as thrombocytopenia. For instance, the introduction of drugs targeting specific oncogenic pathways has been associated with varying degrees of hematological toxicity, including platelet depletion. Recent studies have highlighted the intricate relationship between novel targeted therapies and thrombocytopenia. Furthermore, the mechanisms underlying this relationship often involve the disruption of megakaryocyte function or direct toxicity to bone marrow, which is crucial for platelet production. In particular, therapies for metastatic breast cancer and non-small cell lung cancer have shown a correlation with significant drops in platelet levels, necessitating careful monitoring of blood counts during treatment (86).

Thrombocytopenia is a common adverse effect associated with various targeted therapies, including PARP inhibitors, EGFR inhibitors, and CDK4/6 inhibitors. A meta-analysis of randomized controlled trials involving PARP inhibitors indicated a significant increase in the risk of thrombocytopenia, with an incidence rate of 2.54 (RR, 2.54; 95% CI, 1.87-3.45; p < 0.00001) compared to control groups. Notably, the risk of high-grade thrombocytopenia (grade ≥3) was also elevated (RR, 2.76; 95% CI, 1.83-4.16; p < 0.00001) (87). In the context of EGFR inhibitors, studies have shown that agents like cetuximab and erlotinib can lead to thrombocytopenia, particularly in patients with pre-existing conditions or those receiving concomitant therapies (88). Meanwhile, CDK4/6 inhibitors, such as palbociclib, ribociclib, and abemaciclib, have been associated with varying degrees of hematologic toxicity, including thrombocytopenia, with palbociclib showing a higher incidence compared to the others (89, 90). A systematic review indicated that 51.1% of patients receiving CDK4/6 inhibitors experienced hematological toxicity, with thrombocytopenia being reported in 2.1% of cases (91). These findings highlight the prevalence of thrombocytopenia as a significant concern in patients undergoing targeted therapy, necessitating vigilant monitoring and management strategies.

Poly ADP-ribose polymerase (PARP) inhibitors are a class of anti-cancer drugs that target PARP and play an important role in the treatment of hereditary cancers with the same “rogue gene,” such as breast cancer, ovarian cancer, prostate cancer, and pancreatic cancer (92–95). PARP1 is expressed in the megakaryocytic lineage and is involved in regulating the differentiation of bone marrow cells. PARP inhibitors affect the proliferation and maturation of megakaryocytes by inhibiting PARP1, thereby reducing the production of platelets (96). Inhibitors (e.g.,niraparib, olaparib) trap PARP1 at DNA damage sites, block repair pathways and trigger the accumulation of DNA breaks, thereby suppress the differentiation and maturation of CD34^+^ bone-marrow progenitors into megakaryocytes, reducing the formation of polyploid megakaryocytes and markedly decrease proplatelet formation and platelet release (97). Thrombocytopenia usually occurs in the first month of treatment and gradually shows a trend of recovery in the second or third month, with most cases being mild to moderate, that is, grades 1 to 2 (95). The combination of targeted therapies with chemotherapy has further implications for the risk of thrombocytopenia. Studies have demonstrated that the concurrent use of PARP inhibitors with chemotherapy regimens can exacerbate the incidence of hematological toxicities, including thrombocytopenia. Despite potential responsiveness, patients receiving PARP inhibitors alongside chemotherapy experienced a significant higher rate of grade 3 thrombocytopenia compared to those receiving chemotherapy or PARP inhibitors alone (98). This synergistic effect may be attributed to the myelosuppressive nature of both treatment modalities, which can lead to compounded risks of cytopenia. Moreover, the use of CDK4/6 inhibitors in combination with endocrine therapy has been shown to improve progression-free survival but also results in a higher incidence of adverse events, including thrombocytopenia (99, 100). Therefore, understanding the incidence of thrombocytopenia associated with these targeted therapies, especially in combination with chemotherapy, is crucial for optimizing treatment regimens and improving patient outcomes.

The incidence of thrombocytopenia associated with ADCs, particularly trastuzumab emtansine (T-DM1) and trastuzumab deruxtecan (T-DXd), has been a focus of recent clinical studies. For instance, a systematic review revealed that T-DM1 was linked to a higher incidence of thrombocytopenia, with reported rates of grade 3 or higher thrombocytopenia reaching approximately 28.1% in TKIs treatment failed breast cancer patients (101). Furthermore, a retrospective cohort study highlighted that patients of Asian ancestry experienced a significantly higher incidence of dose adjustments due to thrombocytopenia when treated with T-DM1 or T-DXd, suggesting potential genetic predispositions that warrant further investigation (102). In clinical practice, the management of thrombocytopenia in patients receiving ADCs often necessitates dose modifications or supportive therapies, such as TPO-RAs, to mitigate treatment interruptions and enhance patient safety (88).

The mechanism by which PARP inhibitors induce thrombocytopenia primarily involves their impact on megakaryocyte differentiation and function. Research indicates that the inhibition of PARP activity can disrupt the signaling pathways essential for megakaryocyte maturation, leading to impaired platelet production. This effect is particularly pronounced in patients with pre-existing low platelet count or those undergoing combination therapies that exacerbate myelosuppression (103). Additionally, ADCs, which deliver cytotoxic agents directly to tumor cells, can inadvertently affect normal hematopoietic cells, including megakaryocytes, resulting in diminished platelet output (104). By delivering cytotoxic agents directly to cancer cells, ADCs can inadvertently affect normal hematopoietic cells, including megakaryocytes. The cytotoxic payload can lead to cell death in these progenitor cells, resulting in reduced platelet production. Additionally, the immune response elicited by ADCs can further contribute to thrombocytopenia through mechanisms such as increased phagocytosis of platelets by activated macrophages (105). The interplay between these therapies and the immune system also plays a crucial role. For example, in ITP, the presence of auto-antibodies against platelets can lead to their premature destruction, a complication that may be exacerbated by certain cancer therapies. Furthermore, the activation of immune pathways by these drugs can lead to an increase in macrophage-mediated clearance of platelets, thereby contributing to thrombocytopenia (106, 107). Moreover, the role of the tumor microenvironment in modulating these effects cannot be overlooked. Tumors can secrete various cytokines that influence hematopoiesis and megakaryocyte function, potentially exacerbating the thrombocytopenic effects of both PARP inhibitors and ADCs (108).

## 4 Current status of traditional treatment methods

### 4.1 Indications and limitations of platelet transfusion

Platelet transfusion remains a critical intervention for managing thrombocytopenia, particularly in patients undergoing chemotherapy, those with hematological malignancies, or individuals with severe bleeding disorders. The primary indications for platelet transfusion include active bleeding, planned surgical procedures, and prophylaxis against bleeding in patients with severely low platelet count (109). Platelet transfusion can effectively reduce the risk of bleeding and mortality, which is the fastest and most effective way to treat severe thrombocytopenia, but it also has risks and complications. For instance, patients may have an allergic or febrile reaction to platelet products. Platelet transfusions may exacerbate thrombosis and should be approached with caution. Platelet transfusions can lead to alloimmunization, where the recipient develops antibodies against donor platelets, resulting in transfusion rejection. This condition complicates further injection and may necessitate the use of HLA-matched platelets, which are not always available (110, 111). Additionally, the risk of transfusion-related acute lung injury (TRALI) and other adverse reactions further complicates the use of platelet transfusions. Transfusions of components containing platelet antibodies may trigger acute and severe thrombocytopenia with transfusion reactions and bleeding (112). Recent studies indicate that the effectiveness of platelet transfusions can be influenced by various factors, including the storage conditions of platelets and the presence of underlying conditions in the recipient, such as sepsis or disseminated intravascular coagulation (DIC).The activity of platelets is greatly affected by temperature, and the optimum storage temperature is 22°C ± 2°C.The shelf life of platelets at room temperature is only 3–7 days. Maintaining an adequate supply of platelets requires a lot of resources, and platelets stored at room temperature are prone to bacterial contamination, which may lead to infection and even sepsis (113).

### 4.2 Application and effects of interleukin-11

Interleukin-11 (IL-11) has emerged as a therapeutic cytokine in the treatment landscape for various hematological disease conditions. Its application primarily revolves around its ability to stimulate megakaryocyte and platelet production, making it a potential effective agent for thrombocytopenia. This cytokine has been shown to enhance platelet recovery in patients suffering from CIT, thereby reducing the incidence of severe thrombocytopenia and its associated complications. Research has demonstrated that rhIL-11 can effectively shorten the duration of thrombocytopenia and increase platelet count, making it a valuable option in the therapeutic arsenal against CIT (114).

#### 4.2.1 Mechanisms of rhIL-11

RhIL-11 is a cytokine that plays a significant role in various biological processes, particularly in hematopoiesis and immune regulation. It is primarily known for its ability to stimulate thrombopoiesis, which is the production of platelets from megakaryocytes in the bone marrow. The therapeutic application of rhIL-11 has been particularly highlighted in the context of CIT, where it aids in the recovery of platelet count in patients undergoing cancer treatments that adversely affect bone marrow function. The mechanism by which rhIL-11 exerts its effects involves the activation of specific signaling pathways that promote megakaryocyte proliferation and maturation, ultimately leading to increased platelet production.Additionally,rhIL-11 has been implicated in other physiological processes such as inflammation and tissue repair, showing its multifaceted role in human health (115).

#### 4.2.2 Biological characteristics of rhIL-11

Interleukin-11 (IL-11) is secreted by human bone marrow stromal cells and interstitial cells, which can directly stimulate the proliferation of hematopoietic stem cells and megakaryocyte progenitors. The biological characteristics of rhIL-11 are crucial for understanding its therapeutic potential. RhIL-11 is a glycoprotein that belongs to the IL-6 cytokine family, exhibiting structural and functional similarities with other interleukins. It is produced in recombinant form primarily using Escherichia coli, which poses challenges in terms of expression levels and the formation of inclusion bodies (116). Recent technical advance has enabled the efficient production of tag-free rhIL-11,enhancing its biological activity and structural integrity. The purified rhIL-11 demonstrates a compact and ordered structure, which is essential for its biological function. *In vitro* studies have shown that rhIL-11 can stimulate the proliferation of hematopoietic progenitor cells, indicating its role in enhancing platelet production and supporting the recovery of patients with thrombocytopenia (117). It enhances the maturation of these cells and promotes the shedding of platelets into the circulation. Moreover, rhIL-11 has been shown to modulate the immune response, further emphasizing its importance in therapeutic applications (118).

#### 4.2.3 Application of rhIL-11 in tumor treatment

RhIL-11 has emerged as a promising therapeutic agent in the management of CIT, a common complication in cancer treatment. Clinical trials have demonstrated the efficacy of rhIL-11 in enhancing platelet count and mitigating adverse effects associated with low platelet levels. A systematic review involving ten randomized controlled trials indicated that rhIL-11 significantly reduced the recovery time of platelet count and decreases the volume of platelet transfusion required in patients with acute leukemia undergoing chemotherapy (114). Furthermore, real-world studies have corroborated these findings, showing that rhIL-11 treatment can provide comparable results in platelet recovery when compared to rhTPO, albeit with lower associated treatment costs (119). In solid tumors, rhIL-11 has been demonstrated to improve platelet count effectively, thus facilitating the continuation of chemotherapy regimens without significant delays or interruption. Studies have also indicated that rhIL-11 not only enhances platelet recovery but also reduces the incidence of bleeding complications associated with low platelet count (120, 121).

#### 4.2.4 Adverse reactions and safety management of rhIL-11

RrhIL-11 is known as a therapeutic agent in treating conditions such as ITP and CIT. However, its use is associated with several adverse reactions that needs attention. Clinical studies have indicated that rhIL-11 can lead to significant cardiovascular effects, including elevated serum brain natriuretic peptide (BNP) levels, which may indicate heart strain or failure. Furthermore, patients receiving rhIL-11 have reported instances of edema and weight gain, alongside fluctuations in blood pressure, which necessitates careful monitoring during treatment (122). In hepatocyte cell lines,rhIL-11 also induces secretion of acute phase proteins (ferritin, fibrinogen,C-reactive protein, haptoglobin, and hemopexin) *in vitro*. In consideration of rhIL-11’s anti-inflammatory effects and protection from epithelial damage,rhIL-11 treatment may ameliorate systemic inflammatory diseases and stimulate epithelial wound healing (123).

### 4.3 RhTPO in the treatment of thrombocytopenia in anti-cancer therapy

#### 4.3.1 Pharmacological effects of rhTPO

Thrombopoietin (TPO) is the most important regulator of megakaryocyte proliferation, differentiation, maturation, and platelet production. It can act on primitive hematopoietic stem cells and cooperate with erythropoietin (EPO) and granulocyte colony-stimulating factor and has been demonstrated to stimulate the recovery of platelet levels and promote the reconstitution of hematopoietic function (124). RhTPO is a critical biological drug widely utilized in the treatment of various types of thrombocytopenia. Its pharmacological effects are primarily attributed to its ability to stimulate megakaryocyte proliferation and enhance platelet production in the bone marrow by binding to the thrombopoietin receptor, which activates intracellular signaling pathways critical for hematopoiesis. The downstream signaling cascades including the Janus tyrosine Kinase and Signal Transducer and Activator of Transcription (JAK/STAT) pathway, Phosphatidylinositol 3-Kinase/Akt (PI3K/Akt) pathway, and the mitogen-activated protein kinase (MAPK) pathway. These pathways collectively promote the survival, proliferation, and differentiation of megakaryocyte progenitor cells in the bone marrow (33, 125). By promoting the proliferation and differentiation of hematopoietic stem cells into megakaryocytes, rhTPO plays a pivotal role in increasing platelet count, thereby mitigating the risks associated with low platelet levels, such as bleeding and improves treatment tolerability.

#### 4.3.2 Clinical research on the application of rhTPO in tumor treatment

In a phase I and II clinical trial, the addition of rhTPO to chemotherapy regimen demonstrated a marked restoration of platelet count and a sustained platelet recovery response (126). The role of rhTPO in patients receiving cytarabine has been investigated and prophylactic administration of thrombopoietin reduced the incidence and duration of thrombocytopenia and platelet transfusion rate, indicating the beneficial use of rhTPO before intensive chemotherapy (127). Professor Vadhan-Raj earlier proposed the importance of early application of rhTPO to alleviate early thrombocytopenia caused by chemotherapy (128). Subsequently, several clinical studies evaluating rhTPO’s efficacy in the treatment of CIT were also conducted (127, 129, 130). The results suggest that prophylactic use of rhTPO can reduce the degree and duration of thrombocytopenia and promote early platelet recovery in patients with solid tumors after chemotherapy without causing serious adverse effects. A multi-center cross-sectional study indicated that rhTPO was frequently used among patients experiencing thrombocytopenia due to targeted therapies and immunotherapy, with outcomes showing comparable platelet recovery rates across different thrombopoietic agents (131).

### 4.4 Thrombopoietin receptor agonist serves as a promising therapy in thrombocytopenia

**Figure 2:**
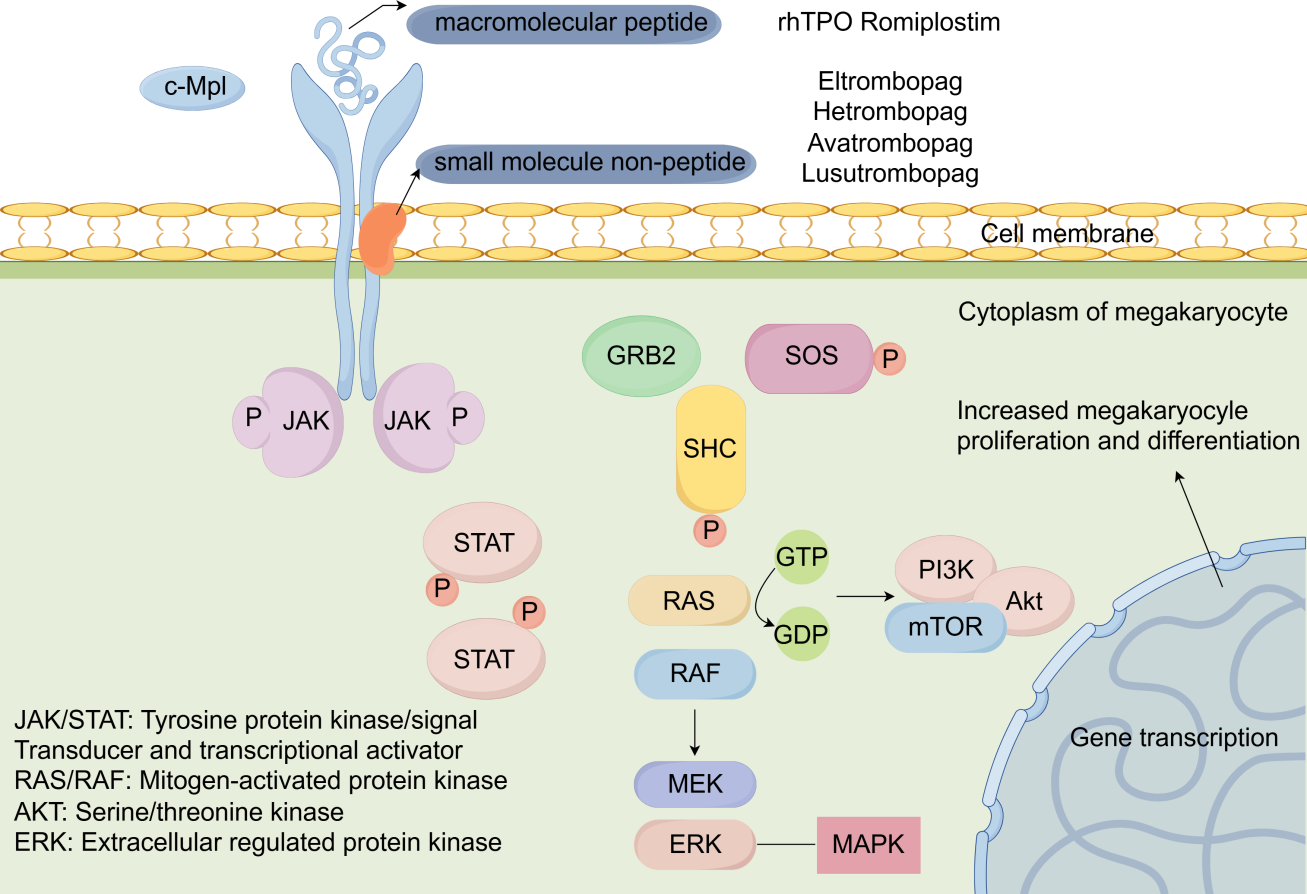
Cellular mechanisms of activation of TPO-RAs.

TPO plays a vital role in the regulation of platelet production and is primarily produced by liver and works on the thrombopoietin receptor (TPO-R), which is expressed on megakaryocytes and hematopoietic stem cells. Cellular mechanisms of activation of TPO-RAs are shown in **Figure 2**. TPO activates JAK/STAT and Ras/MAPK signaling pathways by binding to the surface receptor and induces tyrosine phosphorylation of a variety of proteins, including JAK2 and STAT5 proteins, thereby activating megakaryocytes and promoting their proliferation and differentiation into platelets (132). This process is essential for maintaining normal platelet count, which is crucial for hemostasis and the prevention of bleeding disorders. In the context of cancer treatment, the disruption of this process can ultimately lead to CTIT (133). Thrombopoietin (TPO) receptor agonists have emerged as a novel therapeutic approach to mitigate this adverse effect, playing vital pharmacological roles in enhancing platelet count. These agents mimic the action of endogenous TPO by stimulating the TPO-R, thereby being increasingly recognized as a crucial component of supportive care in cancer treatment.

A network meta-analysis indirectly evaluated the effects of five TPO-RAs in treating ITP. The analysis included 20 RCTs with data from 2,207 patients. All TPO-RAs significantly improved platelet response and reduced the incidence of bleeding events. compared to placebo. Avatrombopag and lusutrombopag showed the best platelet increase response, while avatrombopag having a non-significant advantage over lusutrombopag. Lusutrombopag had the lowest risk of bleeding compared to placebo, followed by eltrombopag, romiplostim, and avatrombopag. Romiplostim had the lowest rate of severe adverse events, followed by avatrombopag, eltrombopag, and lusutrombopag (134).

#### 4.4.1 Eltrombopag

Eltrombopag is a non-peptide thrombopoietin receptor agonist that plays a crucial role in stimulating platelet production in patients with thrombocytopenia. By binding to the thrombopoietin receptor (MPL), eltrombopag mimics the action of endogenous thrombopoietin, resulting in increased megakaryocyte maturation and subsequent release of platelets into the bloodstream (135). This mechanism is beneficial for treating thrombocytopenia associated with various conditions, including ITP, aplastic anemia, and CIT. Recent studies have also indicated that eltrombopag may exert additional effects beyond mere stimulation of megakaryopoiesis. For instance, it has been shown to possess immunomodulatory properties, potentially influencing T-cell responses and reducing inflammatory cytokines, which can contribute to its therapeutic efficacy in various hematological disorders (136, 137).

In a phase 2 clinical trial, it involved patients with solid tumors including non-small cell lung cancer, breast cancer, pancreatic cancer and others, and was conducted to compare the efficacy of eltrombopag with placebo. The results showed that the mean time for platelets to recover from nadir was 8 days in the eltrombopag group and 15 days in the placebo group. Patients in the eltrombopag treatment group had fewer platelet count below 100×10^9^/L, fewer doses modification, and fewer adverse events (138). A real-world observational study compared eltrombopag with rhTPO and found that both agents had comparable efficacy and safety in terms of increased platelet count, prolonged platelet response time, reduced bleeding events, and reduced need for platelet transfusions in lymphoma patients experiencing severe thrombocytopenia following chemotherapy (139). However, it should be noted that the higher the highest platelet count after eltrombopag treatment, the greater the likelihood of side effects. The reported incidence of treatment-related adverse events has been relatively low, with studies indicating that only 12-14% of patients experience mild and transient adverse reactions, including headache (21.6%), asthenia (13.7%), hepatotoxicity (11.8%), and thrombosis (5.9%) (139, 140).

#### 4.4.2 Hetrombopag

Hetrombopag is developed through structural modifications of eltrombopag, which are designed to enhance its potency while reducing toxicity. On 16 June 2021,Hetrombopag was first approved in China for treating adult patients with primary ITP who had shown poor response to prior treatments like glucocorticoids and immunoglobulins and was conditionally approved for the treatment of severe aplastic anemia (SAA) in patients refractory to immunosuppressive therapy. Hetrombopag stimulates intracellular thrombopoietin signaling pathways (including STAT3, STAT5, ERK1/2 and AKT) and promotes megakaryocyte proliferation and differentiation in proportion to concentration (141).

Results from recent clinical trials have shown promising efficacy for hetrombopag (142–144), with significant improvements noted in enhanced responses in patients with specific cancer types, underscoring the drug’s potential as a valuable therapeutic option, particularly in oncology. For instance, hetrombopag significantly improved the condition of low platelet count after chemotherapy in patients with advanced solid tumors in a randomized, placebo-controlled phase II study. The response rate of the comparative groups was 60.7% (17/28) versus 12.9% (4/31) respectively. Most commonly observed adverse events were decreased neutrophil counts and anemia (145). Hetrombopag showed synergistic effect in stimulating platelet production when in combination with rhTPO, necessitating an effective management of thrombocytopenia. The proportion of recovery patients was higher than rhTPO alone, there was no significant difference in the incidence of adverse events between the two groups which involved bleeding, platelet transfusion, elevated liver enzymes, hyperbilirubinemia, fever, fatigue, diarrhea (146). In consistence with this clinical advantage, hetrombopag combined with rhTPO generated proliferation-promoting effect and anti-apoptotic effect in TPOR-expressing cells through stimulation of STAT, PI3K and ERK signaling pathways (141).

#### 4.4.3 Romiplostim

Romiplostim is produced in Escherichia coli by recombinant DNA technology a recombinant fusion protein containing two identical single-chain subunits, each of which is covalently linked by a Fc domain of human immunoglobulin IgG1 and a short peptide with high affinity for the TPO-R consisting of 14 amino acids (147, 148). Romiplostim has no sequence homology to endogenous TPO and stimulates the proliferation and differentiation of megakaryocytes through the binding and activation of TPO-R and stimulation of C-Mannosylation of thrombopoietin(c-Mpl),JAK2-STAT5,PI3K/Akt, MEK/ERK, and p38 downstream signaling pathways, thereby producing pharmacological efficacy (147). Romiplostim is prevented from being cleared by the renal route when bound to the c-Mpl receptor. Preclinical study indicated that romiplostim not only increased platelet count but also had immunomodulatory effects by lowering anti-platelet antibody levels (149, 150). Romiplostim is also considered an alternative treatment option for patients who respond negative to rhIL-1 or rhTPO.

Soff et al. (151) reported a randomized controlled prospective trial comparing the efficacy of romiplostim and observation in patients with CIT from solid tumors. The therapeutic regimen was altered because romiplostim significantly increased the proportion of patients with platelet recovery within 3 weeks of treatment compared to the observation group (93% vs 12.5%). All patients in the observation group were subsequently crossed over to the romiplostim treatment group and continued to receive romiplostim prophylaxis for CTIT during subsequent chemotherapy and a similar response rate was observed. Al-Samkari et al. (152) performed a multi-center study including 173 patients across four US centers, with a focus on assessing the effectiveness and safety of romiplostim, as well as identifying predictors of non-response. The study found that 71% of solid tumor patients achieved a romiplostim response (defined as a median platelet count≥75×10^^9^/L and ≥30×10^^9^/L above baseline while on treatment),the median platelet count on romiplostim was significantly higher than baseline (116×10^^9^/L vs. 60×10^^9^/L; p<0.001) and 79% of solid tumor patients avoided chemotherapy dose reductions or treatment delays, and 89% avoided platelet transfusions. Bone marrow (BM) invasion by tumor, prior pelvic irradiation, and prior exposure to temozolomide were identified as significant predictors of romiplostim non-response. Patients with these predictors had lower response rates (23%, 20%, and 46% respectively).Weekly romiplostim dosing was superior to intracycle dosing in terms of higher response rates (81% vs. 63%) and fewer chemotherapy dose reductions/treatment delays (IRR 3.00, P = 0.010) and bleeding events (IRR 4.84, P = 0.029).In patients with non-myeloid hematologic malignancies(lymphoma and myeloma), the response rate was lower (10%), with only 35% achieving a platelet count ≥100×10^9/L. The median platelet count improved from 21×10^9/L at baseline to 46×10^9/L on romiplostim.

Refractory CTIT is generally defined as: platelet count remains <50 × 10^9^/L or cannot be sustained ≥50 × 10^9^/L after an adequate dose and duration (≥4–6 weeks) of TPO-RA therapy (romiplostim or eltrombopag); or ongoing reliance on platelet transfusions for support (43, 153). Real-world data show that romiplostim non-responders have a chemotherapy relative dose intensity (RDI) below 60% within six months and a 25% reduction in overall survival (OS) (152). Tailored escalation of TPO-RAs or combination strategies coupled with investigations into stem-cell or megakaryocyte replacement therapies constitutes the central management paradigm.30–40% of patients achieve a secondary response by switching TPO-RAs (154). Combining low-dose decitabine (3-day cycles) or low-dose cyclosporine A (1–2 mg/kg) can overcome immune barriers (155).

#### 4.4.4 Lusutrombopag

Lusutrombopag is a small-molecule thrombopoietin receptor agonist(TPO-RA) that has gained attention for its potential use in managing thrombocytopenia, including CTIT. Lusutrombopag has a high affinity for the TPO receptor and is designed to specifically target this receptor without cross-reactivity to other receptors, minimizing off-target effects. Lusutrombopag activates downstream signaling pathways, such as the JAK2-STAT pathway. Lusutrombopag is primarily metabolized in the liver through enzymatic processes. The metabolites are then excreted through both renal and biliary pathways (156, 157). Lusutrombopag has received marketing authorization in Japan, the United States, and Europe for thrombocytopenia associated with chronic liver disease and platelet elevation therapy prior to elective invasive surgery or diagnostic procedures.

Sato et al. (157) reported the efficacy of lusutrombopag in patients with hepatocellular carcinoma who underwent both radiofrequency ablation and transarterial chemoembolization. The patients’ platelet count increased to 98 × 10^9^/L after 5 consecutive days of lusutrombopag treatment. After lusutrombopag usage within 7 days after transcatheter arterial chemoembolization, the platelet count reached more than 50 × 10^9^/L. Ishikawa et al. (158) reported the efficacy and safety of retreatment with lusutrombopag before multiple planned radiofrequency ablation in 8 patients with hepatocellular carcinoma and thrombocytopenia. The results of the study showed that repeated use of lusutrombopag did not reduce its effect on increasing platelet count. The drug significantly reducing the need for chemotherapy dose reductions, treatment delays, and platelet transfusions. Lusutrombopag has demonstrated a favorable safety profile in clinical trials. Common side effects are generally mild and may include gastrointestinal symptoms such as nausea and diarrhea. Serious adverse events, such as thromboembolic events, have been reported at low rates, but they need to be carefully monitored, especially in high risk populations (159).

#### 4.4.5 Avatrombopag

Avatrombopag is an orally bioavailable small molecule TPO-RA. Avatrombopag does not compete with TPO for binding to the TPO receptor and has an additive effect with TPO on platelet production. Avatrombopag is primarily metabolized through the cytochrome P450 (CYP) enzyme system, with CYP2C9 and CYP3A being the main isoenzymes involved. Avatrombopag is > 96% bound to human plasma proteins and has extensive distribution according to *in vitro* data (160). It is the third TPO-RA approved for the treatment of ITP and the first approved for the treatment of perioperative thrombocytopenia in patients with chronic liver disease. Avatrombopag provides an alternative to blood transfusion in these patients. Unlike eltrombopag, the use of avatrombopag does not require a 4-hour window of caloric restriction and does not have the associated problems of hepatotoxicity in patients with ITP or portal vein thrombosis in patients with chronic liver disease (161).

Results of a phase 3 randomized controlled trial of the efficacy and safety of avatrombopag in CIT have been published for better understanding the benefits of avatrombopag in clinical setting (162). This international, randomized, double-blind, placebo controlled, phase 3 study enrolled 122 patients aged 18 years or older with ovarian, bladder, or lung cancer receiving chemotherapy and experiencing severe thrombocytopenia (platelet count <50 × 10^9^ cells/L). Patients were randomized into 2:1 to receive either avatrombopag 60 mg or placebo orally daily for 5 days before and after chemotherapy. Only 69.5% of patients treated with avatrombopag met the primary endpoint of avoiding platelet transfusion, reducing the dose of chemotherapeutic agents, and delaying administration, compared with 72.5% of patients treated with placebo. However, avatrombopag increased the lowest platelet count compared to placebo (51×10^9^cells/L vs. 29.1×10^9^ cells/L).Avatrombopag was safe and well-tolerated, with no increased incidence of thromboembolic events. The study suggests that further investigation of avatrombopag in patients with more persistent CIT might be required to confirm its ability to increase platelet count.

Recently, the potential benefits of combining TPO receptor agonists with platelet transfusion in managing CIT have been reported (163). The study retrospectively collected clinical data from 120 patients with malignant tumors who developed thrombocytopenia following chemotherapy. Patients were randomly divided into three groups: Group A received avatrombopag, Group B received autologous platelet transfusion, and Group C received a combination of both treatments. The study found that Group C (combination treatment) achieved the highest effective rate of 97.50%,significantly higher than Group A (avatrombopag alone) at 80.00% and Group B (autologous platelet transfusion alone) at 77.50% (P < 0.05). The time for platelet count recovery was significantly shorter in Group C. Group C had significantly higher levels of PF4, TPO, and vWF compared to Groups A and B (P < 0.05). Post-treatment levels of APTT and PT decreased in all groups, with Group C showing significantly lower APTT compared to Groups A and B (P < 0.05). In conclusion, the combined therapy resulted in faster platelet recovery, higher platelet count, and improved coagulation function indicators.

**Figure 3:**
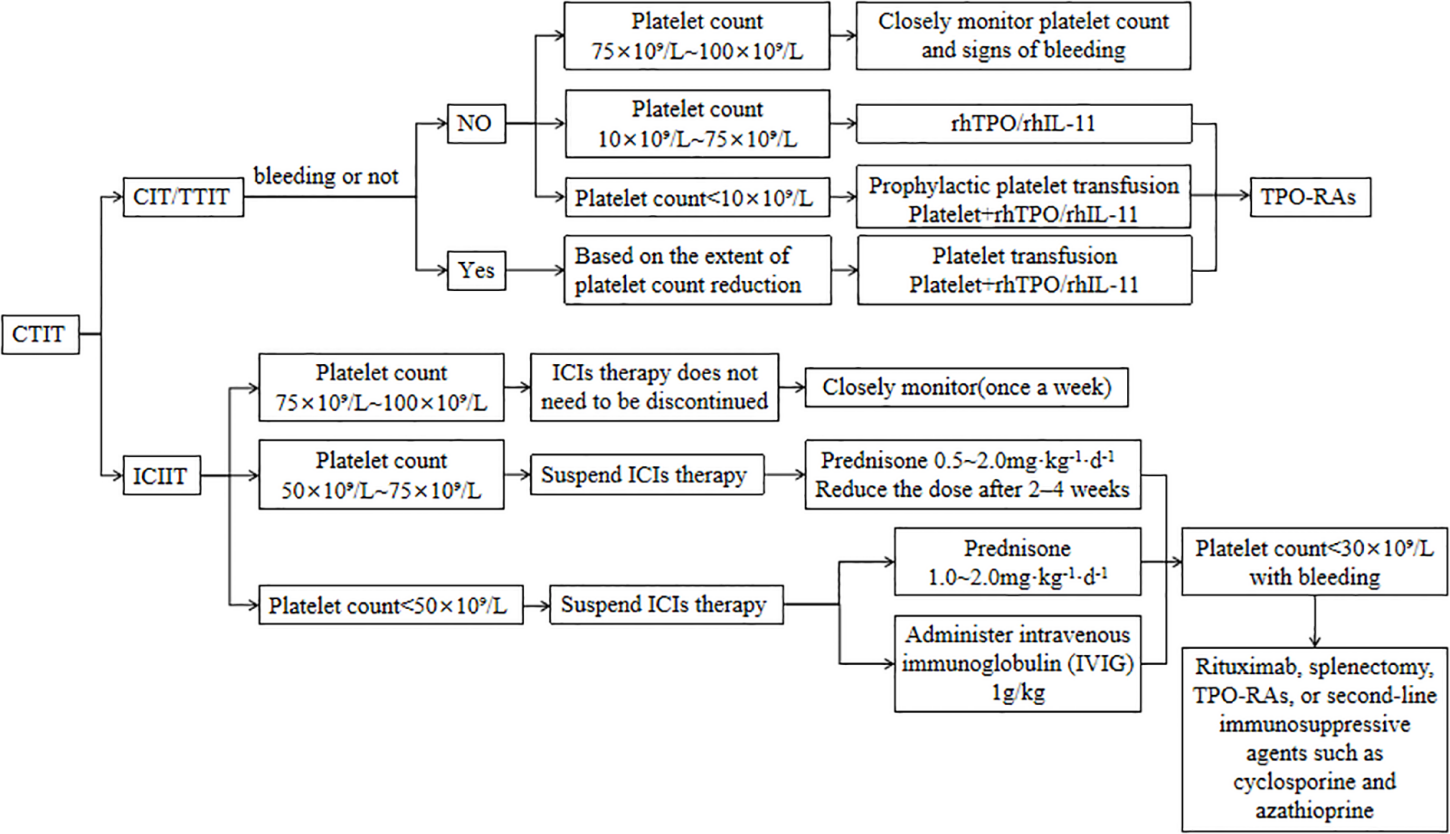
Therapeutic algorithm for CTIT.

CTIT management should first clarify the etiology, assess bleeding risk, and then tailor the therapeutic strategy according to the underlying cause and CTIT severity; the main interventions are platelet transfusion and administration of thrombopoietic growth factors. While administering thrombopoietic agents, closely monitor for adverse reactions, promptly manage any events that occur, and thereby maximize treatment safety and efficacy. Grade 3–4 thrombocytopenia warrants immediate treatment discontinuation, whereas patients on long-term antineoplastic therapy who develop grade 1–2 thrombocytopenia without bleeding can continue the original regimen under close monitoring. Therapeutic algorithm for CTIT is shown in **Figure 3**.

### 4.5 Application of caffeic acid tablets in CTIT

Caffeic acid tablets, an emerging therapeutic agent, primarily composed of caffeic acid which was originally found in coffee extracts and structurally belongs to hydroxycinnamic acid compounds (164). Caffeic acid have been shown to promote megakaryocyte maturation and enhance platelet production. As an antioxidant, caffeic acid effectively neutralizes free radicals, thereby protecting cells from oxidative damage, which is a contributing factor in various chronic diseases, including cancer and cardiovascular disorders (165–167). *In vitro* studies demonstrated that the combination of caffeic acid and hydroxydaunorubicin (DOX) exhibits a significant inhibitory effect on lung cancer cells. Caffeic acid modulates the proliferation of lung cancer cells via the MAPK signaling pathway (165).

A total of 35 publications with 2533 patients were included in a meta-analysis study to evaluate the efficacy and safety of caffeic acid tablets (CFA). The study showed that CFA significantly increased platelet counts compared to the control group. CFA demonstrated better clinical efficacy in treating thrombocytopenia, with complete remission defined as PLT≥100× 10^^9^/L and partial remission as 50×10^^9^/L < PLT < 100×10^^9^/L.CFA also significantly increased white blood cell counts, neutrophil counts and reduced the incidence of grade III and IV myelosuppression (167). In hepatocellular carcinoma cells, caffeic acid inhibited the expression of vascular endothelial growth factor (VEGF) and matrix metalloproteinase 9 (MM-9) which induces tumor invasiveness and metastases (168). Caffeic acid predominantly suppresses the growth of estrogen receptor (ER) positive breast cancer cells (169).

### 4.6 Traditional Chinese medicine

The application of traditional Chinese medicine (TCM) in the treatment of cancer is gradually gaining attention, especially in terms of modulating immune responses, improving patients’ quality of life, and reducing side effects (170). The therapeutic mechanisms of TCM are primarily reflected in its multi-component and multi-target characteristics, which sharply contrast with the single-target drug design in modern medicine. TCM can be used as an adjuvant therapy to enhance the efficacy of conventional cancer treatments (170). Chemotherapy often causes severe side effects, including nausea, vomiting, myelosuppression, and organ dysfunction. TCM can help alleviate these adverse reactions (171, 172). For instance, a systematic review and meta-analysis involving 22 randomized controlled trials (RCTs) with 1834 postoperative breast cancer patients revealed that TCM combined with chemotherapy significantly improved therapeutic efficacy, immune function, and reduced adverse reactions such as leukopenia and thrombocytopenia (173). While some RCTs suggest benefits, evidence remains preliminary due to methodological heterogeneity and lack of large-scale trials. A study found that combining Traditional Chinese Medicine (TCM) with capecitabine-based chemotherapy in colorectal cancer (CRC) is more effective than capecitabine alone. The combination therapy enhances tumor response rates and reduced the risks of thrombocytopenia (174). Moreover, a network meta-analysis highlighted the efficacy of various TCM injections, such as Aidi and Compound Kushen injections, in improving treatment responses and reducing adverse effects when used in conjunction with chemotherapy in colorectal cancer patients (175).

## 5 Discussion

In summary, CTIT is a prevalent adverse effect of anti-tumor therapies that significantly impacts treatment efficacy and patients’ quality of life. The treatment and clinical research of CTIT has emerged as a significant concern in the realm of oncology. Platelet transfusion is the basic treatment for CIT, and the widespread use of rhIL-11 and rhTPO has significantly reduced the number of patients with chemotherapy restrictions, thus greatly reducing the need for platelet transfusion. While rhIL-11 has been approved by the FDA for use in CIT, its use has been limited due to its side effect, and manufacturers have stopped offering the drug. However, rhTPO, which is not associated with cross-reactive antibodies, completed its development in China and has become a routine component of supportive care for cancer patients. Compared with other TPO-RAs, rhTPO has lower efficacy and more complications, but with the increase of clinical trial data, TPO-RAs have become a reliable choice for the treatment of CTIT.

This review aims to explore the etiology of CTIT, its implications on patient outcomes, and the current treatment and preventive measures available. By synthesizing existing literature, we seek to provide clinicians with comprehensive management strategies to mitigate bleeding risks and enhance patients’ therapeutic effect during cancer treatment. In addition, by integrating genomic, proteomic, and metabolic insights into the development of personalized treatment protocols, we can better predict and manage the risks associated with cancer therapies. It is important to emphasize personalized treatment strategies and preventive measures tailored to each patient’s biological characteristics, genetic profile, and drug response.
